# Enhancing lipid production in *Nannochloropsis salina* via RNAi-mediated downregulation of carbohydrate biosynthesis

**DOI:** 10.3389/fmicb.2025.1601691

**Published:** 2025-05-21

**Authors:** Hyun Gi Koh, Kyungmoon Park, See-Hyoung Park, Minsik Kim, Nam Kyu Kang

**Affiliations:** ^1^Department of Biological and Chemical Engineering, Hongik University, Sejong, Republic of Korea; ^2^Department of Biological Engineering, Inha University, Incheon, Republic of Korea; ^3^Department of Chemical Engineering, College of Engineering, Kyung Hee University, Yongin, Republic of Korea

**Keywords:** microalgae, *Nannochloropsis salina*, RNAi, UGPase, carbohydrate, lipid

## Abstract

Microalgae are promising platforms for sustainable biofuel production owing to their high photosynthetic efficiency and carbon fixation capacity. *Nannochloropsis salina* is particularly valued for its robust growth and lipid accumulation. However, redirecting carbon flux from carbohydrate to lipid biosynthesis remains a key challenge in microalgal metabolic engineering. In this study, RNA interference (RNAi) was employed to downregulate uridine diphosphate-glucose pyrophosphorylase (UGPase), a central enzyme in chrysolaminarin biosynthesis. After confirming the presence of core RNAi machinery (Argonaute, Dicer, and RDR) in *N. salina*, an RNAi construct targeting UGPase was introduced. Two transformants, NsRiUGPase 5 and NsRiUGPase 26, were selected through McrBC-PCR and qRT-PCR screening based on reduced methylation-sensitive PCR band intensity and UGPase transcript levels. These RNAi mutants exhibited significantly enhanced growth compared to wild-type. On day 12, dry cell weight (DCW) reached 4.77 g/L in NsRiUGPase 5 and 6.37 g/L in NsRiUGPase 26, representing 32.4% and 76.9% increases, respectively, compared to WT (3.60 g/L). Despite similar lipid contents per biomass, lipid productivity was markedly improved. On day 12, NsRiUGPase 26 achieved 196.3 mg/L/day, a 71.0% increase over WT (114.8 mg/L/day). Fatty acid methyl ester (FAME) analysis showed no significant difference in lipid composition among strains, indicating that UGPase knockdown did not affect lipid quality. These results demonstrate that RNAi-mediated suppression of UGPase successfully redirected carbon flux away from carbohydrate storage toward growth, thereby enhancing overall lipid productivity. This study provides new insights into carbon partitioning in *N. salina* and underscores RNAi as a powerful tool for microalgal biofuel optimization.

## 1 Introduction

Microalgae have emerged as a promising platform for sustainable bioproducts and biofuel production due to their rapid growth, high photosynthetic efficiency, and ability to sequester atmospheric CO2 (Wang et al., 2024). Unlike terrestrial crops, microalgae can be cultivated in non-arable land and wastewater, reducing competition with food production and minimizing environmental impact (Babu et al., 2022). Additionally, their metabolic flexibility enables the production of various valuable compounds, including lipids, carbohydrates, proteins, and pigments (Ranjbar and Malcata, 2022). Given the increasing global demand for renewable energy sources, enhancing the lipid productivity of microalgae has become a key research focus in biotechnology (Banerjee et al., 2018).

Among various microalgal species, *Nannochloropsis* has gained particular attention due to its naturally high lipid content and robust adaptability to diverse cultivation conditions (Liu et al., 2017; Poliner et al., 2018). This species accumulates substantial amounts of neutral lipids, particularly triacylglycerols (TAGs), which are crucial precursors for biodiesel production (Babu et al., 2022). Furthermore, *Nannochloropsis* exhibits high tolerance to environmental fluctuations, making it an ideal candidate for large-scale biofuel production (Bartley et al., 2013). As a result, extensive metabolic engineering efforts have been undertaken to enhance lipid accumulation (Poliner et al., 2018).

Despite significant progress in microalgal metabolic engineering, precise and efficient gene regulation remains a major challenge (Fajardo et al., 2020). This is largely due to the low transformation efficiency, limited availability of molecular tools, and the complexity of metabolic regulatory networks in microalgae (Kang et al., 2022). Recent advances in genome editing technologies, particularly the development of CRISPR/Cas9 system in microalgae, have improved gene targeting and metabolic pathway modifications (Jeon et al., 2017, Muthukrishnan, 2022). However, effective downregulation of specific genes, which is essential for redirecting metabolic flux, remains difficult. Existing genome editing approaches, such as CRISPR/Cas9, are often constrained by inefficient homology-directed repair (HDR) and challenges in mutant screening (V. K. Patel et al., 2019; Shin et al., 2016).

RNA interference (RNAi) is a post-transcriptional gene silencing mechanism that utilizes small RNA molecules to target and degrade specific mRNA sequences, thereby preventing protein synthesis (Agrawal et al., 2003, Rana, 2007). This approach provides a highly specific and reversible method for gene suppression, making it an attractive alternative to CRISPR/Cas9 for metabolic engineering in microalgae (Fajardo et al., 2020). Unlike CRISPR-based gene knockouts, which involve permanent genetic alterations, RNAi allows transient and tunable regulation of target genes without introducing permanent modifications to the genome. Several studies have successfully employed RNAi to enhance lipid accumulation and modify metabolic pathways in microalgae. For example, Ma et al. (2017) used RNAi to silence *pyruvate dehydrogenase kinase (PDK)* in *Nannochloropsis salina*, which redirected carbon flux toward triacylglycerol (TAG) biosynthesis, resulting in increased lipid accumulation (Ma et al., 2017). Similarly, Trentacoste et al. (2013) applied RNAi in *Thalassiosira pseudonana* to downregulate genes involved in lipid catabolism, leading to enhanced lipid storage without compromising growth (Agrawal et al., 2003). Moreover, Li et al. (2019) demonstrated that RNAi-mediated silencing of *CPS* and *UGDH* genes in *Nannochloropsis oceanica* not only promoted lipid overproduction but also enabled lipid secretion, which is critical for biofuel applications (Li et al., 2019).

In this study, we employed RNA interference (RNAi) technology to downregulate uridine diphosphate-glucose pyrophosphorylase (UGPase) expression in *Nannochloropsis salina*, aiming to reduce the accumulation of chrysolaminarin, a storage polysaccharide, and enhance lipid biosynthesis (Vogler et al., 2021; Supplementary Figure 1). By suppressing UGPase, we successfully redirected carbon flux from carbohydrate storage toward biomass and lipid production. Our findings demonstrate that RNAi-mediated gene silencing is a highly effective approach for microalgal metabolic engineering, offering a precise and efficient method to regulate gene expression. This study not only provides new insights into the metabolic regulation of lipid biosynthesis in *Nannochloropsis* but also highlights the potential of RNAi as a powerful tool for optimizing biofuel production in microalgae.

## 2 Materials and methods

### 2.1 Strains and cultivation

Nannochloropsis salina CCMP 1,776, obtained from the National Center for Marine Algae and Microbiota, was cultivated under photoautotrophic conditions in a modified F2N medium (Kang et al., 2015). The medium was prepared with 427.5 mg/L NaNO3, 30 mg/L NaH2PO4⋅2H2O, and 10 mM Tris-HCl (pH 7.6). Additionally, it contained 5 mL of a trace metal solution comprising 4.36 g/L Na2EDTA⋅2H2O, 3.15 g/L FeCl3⋅6H2O, 10 mg/L CoCl2⋅6H2O, 22 mg/L ZnSO4⋅7H2O, 180 mg/L MnCl2⋅4H2O, 9.8 mg/L CuSO4⋅5H2O, and 6.3 mg/L Na2MoO4⋅2H2O. The medium was supplemented with 2.5 mL/L of a vitamin stock solution containing 1 mg/L vitamin B12, 1 mg/L biotin, and 200 mg/L thiamine⋅HCl, along with 15 g/L sea salt (Sigma-Aldrich, MO, United States). The cultures were maintained in 250 mL baffled Erlenmeyer flasks with 200 mL of F2N medium at 25°C under a continuous light intensity of 120 μmol photons/m^2^/s, with orbital shaking at 120 rpm. Air enriched with 2% CO2 was continuously supplied at a rate of 0.5 vvm.

### 2.2 Vector construction and transformation

**FIGURE 1:**
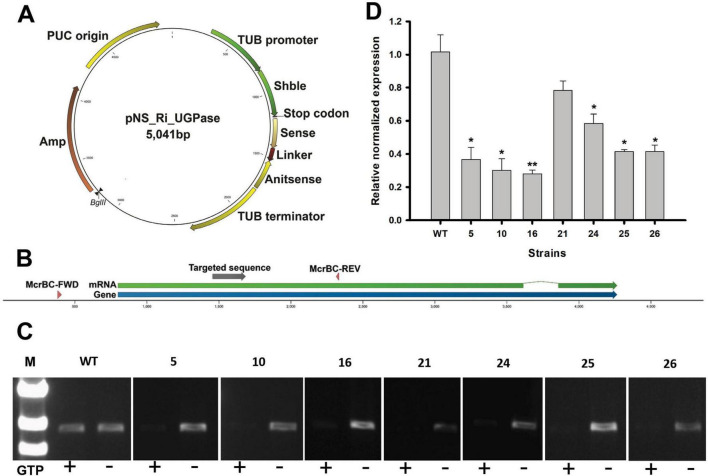
Uridine diphosphate-glucose pyrophosphorylase (UGPase) RNA interference (RNAi) knockdown validation using McrBC-PCR and quantitative real-time PCR (qRT-PCR). **(A)** Schematic representation of the RNAi knockdown vector targeting UGPase. The construct includes a sense and antisense region separated by a linker, driven by the TUB promoter and terminator. The Shble gene provides selection. **(B)** UGPase gene region and the locations of primers used for McrBC-PCR (McrBC-FWD, McrBC-REV) and qRT-PCR (Q1, Q2). **(C)** McrBC-PCR results. In the presence of GTP, methylated DNA is cleaved, leading to weaker PCR amplification. This indirectly suggests successful RNAi-mediated methylation-dependent degradation. **(D)** qRT-PCR analysis of mRNA expression levels in RNAi-targeted regions using Q1 and Q2 primers. The relative expression levels indicate the effectiveness of RNAi-mediated knockdown. The data points represent the average of samples and error bars indicate standard error (*n* = 3). Significant differences against wild-type (WT) for the same conditions and same time points, as determined by Student’s *t*-test, are indicated by asterisks (*p < 0.05, **p < 0.01, ***p < 0.001).

The UGPase coding sequence and the RNAi target region are summarized in Table 1, while the primers used for vector construction are listed in Table 2. The Shble resistance gene and the sense strand of the RNAi cassette were amplified using P1 and P2 primers. The linker, antisense strand, and TUB terminator were amplified with P3 and P4 primers, and the pNs_Ri_UGPase vector backbone was amplified using P5 and P6 primers. These three PCR products were assembled into the final vector using the Gibson assembly technique (Gibson et al., 2009). In the constructed pNs_Ri_UGPase vector, the Shble resistance gene and the RNAi cassette were placed under the control of the endogenous TUB promoter and TUB terminator, ensuring proper expression in N. salina (Figure 1A).

**TABLE 1:** Uridine diphosphate-glucose pyrophosphorylase (UGPase) gene sequence and RNA interference (RNAi) target region.

Name	Sequence (5′ -> 3′)
UGPase coding sequence	ATGCTCGCCTTCCCCACTCCGGGGCTCACGGCCCCTTTCCTCGCCATCACGCAGGCCAAAATGCGGAAAGAGCATCTGAGTGACGCGG CCATTGCCTCCTTCAAAAATTCTTACATGGCCCTGGTGTCGGGCGCCTCAGGAGTCATTGCCGAAAGCGATATCAAACCCGCCGAGGG CCTACCCAATCTGGAGAAAGATCTAAAGCCCAAGATCAAGGTGAACCCGGAGCTTCTCAAGGAAACCGTGGTCTTGAAACTGAACGGC GGGCTGGGCACTGGCATGGGCTTGGACAAGGCGAAATCCCTGCTGCCCGTGAAGGGGAAGGATACCTTCTTGGACCTGACCGCCAAG CAAGTCATGGCCTTCCGGCATAAATTCAAGAGCCACGTCCGCTTTATCCTCATGAACTCCTTCAGCACCTCCGAGGACACGCTCAGCTA CCTGAGCAAGTACCCGGCGTTGGTCGCCGACCCCAACCTGGAGCTCCTGCAAAACAAAGTCCCCAAGGTGGATGCGGCCAGCCTGGA GCCAGTGGCATGGCCCACGAATCCCGCGCAAGAGTGGTGTCCTCCAGGCCACGGCGATCTCTACGCGGCCTTGGACGGATCGGGGAC CTTGGACCGTCTCCTGGCGGACGGGGTCAAGTACATGTTCGTGTCCAATTCGGACAACCTGGGCGCGACCTTGGATCTGTCCCTGCTC ACGTATTTCGCCGAGTCGGGCTCCTCCTTCATGATGGAGTGCGCGGAGAGGACGGAGGCGGACAAGAAGGGGGGGCACCTGGCGGTA CGGTCCAGCGACGGCCAGCTGATCTTGCGGGAATCTGCCCAGTGTGCCAAGGAAGACGAACCCGCCTTCCAAGATGTCTCGCGGCAC AAGTACTTCAACACCAACAACCTGTGGGTGCGCCTGGACAAGCTCAAGGAGGCCACCATCGCCGCCGGGGGTCTCATCCCCCTCCCCA TGATCAAGAACGGGAAGACCGTGGACCCCAAGGACGGGAAAAGCCCCAAAGTTTGGCAGCTGGAGACGGCGATGGGCGCGGCCATT GAATGCTTTCCCGGCTCGTCCGCCGTGGTCGTGCCCCGCACCCGCTTTGTCCCCGTCAAGAAATGCAACGACCTCCTCCTCCTCCGCT CCGACACCTACGTGCTCACTGCCGACGGAACTCCGGCTCTGGACCCGTCTCGGCACGGCGCCGCCCCTCTCATCAATTTGGACGATAAA GCCTACAAGCTGGTGCAGCAGCTCGAGGCGGCCACTCAGGGGGGAACGCCCTCTCTGGTGGGCGCGGACCGCTTGACCATCCAAGGG CAAGTCTGGCTCTCCTCGGGGGTCGTCTTTCAGGGCACCACCACGGTCACGAACACGGGCGACGAGCCCAAAGTGCTGCCGAAGGGG GTTTACAAGGATGCGAGTGTGGACCTGACGGCGGCGCCCGGTCTCGGGGCCCTCCGGCCCACGATTGTGGCCACGGCTCCCATCCCC GGACAAAAGCCTGGTACCTCAGGGCTTAGGAAGAAGGTGGTGGAGTTCCAAAGCCCCCATTACCTGAACAATTTCGTGCAGGCCGTA TTTAATGCCTTGGTGGACTTTGGGACGGACGTGACCCTGGGGGGTAGTCTGGTTGTGGGCGGGGACGGCCGCTACTTTAACCCCGAAG CCATCCAAATCATTACCAAGATGGCGGTGGCCAACGGAGTCAAACGGATCTTGATCGCCAAAGACGGCCTTCTCTCGACCCCAGCCGC GTCGGCGGTCATCCGTGAGCGGGGCCCCGCCTGGCAAAAGGCGTTCGGCGCGTTCATCCTCTCTGCTTCCCACAACCCGGGCGGACC CTCGGAAGACTTTGGGATCAAGTACAACATCGAAAATGGAGGACCGGCTCCCGAGAAGGTGACCAACGCCATGTACTCGTATACCACC ACCCTGACCTCCTACAAAATCGCGCCGGATTTCCCAGATGTGGACACAAGTAAGCTGGGCAGCACCCGGGTCGTCTCGGCGGACGGGA GCCGTGGTGTGGTGGTGACCGTGTTCGACGGCCTGGAGGGGCACGTAAAGCTGCTCAAGACCATCTTCGATTTCGAGGCGATCCAGAC GCTTATGCAACGGCCGGATTTTTCCCTTGTCTACGACTCCATGTCCGGAGTCCAGGGGCCGTATGCCCACAAGGTGTTTGTGGAGGAG TTGGGCGCGGCGACGACCTGTCTCTTGAACGCGGAGCCCAAGGATGATTTCGGGGGAGGGCATGCGGATCCGAATCTGACGTACGC ACACGATCTCATCCATGTGATGGGGGTGGACAGCAAGGGTAACGCGGTGGCTGCGAAGGAGGGGGTCTTGATCCCTTCCTTCGGCGCGG CGGCGGATGGAGACGCGGACAGGAACATGATTTTGGGCCGACAGTTCTTTGTGACGCCCTCGGATTCCTTGGCGATCATCGTGGCGC ACGCAGACGTGATCCCCTTCTTCCGGGACCAGGGAGGCCTGCGTGGGGTCGCGCGGTCCATGCCCACGAGCGGTGCGGTGGACCTGG TGGCCAAGCGGATGAACATGAGCCTGTTCGAGACCCCGACGGGCTGGAAATTCTTTGGAAACCTGATGGACAGCCGGGAGATGGGCG GTGCGAACTACACGCCCTTCATTTGCGGGGAGGAGAGCTTTGGCACGGGCTCGGATCACGTCCGGGAGAAGGACGGCATGTGGGCTG TCCTGGCCTGGCTGTCCATTCTGGCCCATTACAACCAAGACCCGAAGAAGGCCCTGGTCTCGGTGGAAAGCATCGTGCGGGAGCATT GGCGGACGTATGGGAGGAACTACTACGTGCGGTACGACTACGAGGGCGTGGACAAGAGCCGGGCCGAGGCCATGGTCGGGCACATG ACCTCCTCCTTTGCCGCCGTTACCGGGCAACGCCTCGCGGGCGGTTACACGGTGGCGGTTGCCGATGAGTTCCAGTACGTGGACCCC GTGGACGGCTCGGTCTCCTCCCACCAGGGGGTCAGGTACCTGTTCACGGACGGTTCGCGCGTCATTTTCCGCCTCTCGGGCACGGCAG GATCCGGTGCCACGGTCCGCATGTACCTGGAGAAGTACGAGGCCGACCGGAGCAAGCTGGGATCGCACCCTCTGGAGGCCCTAGGCG TCTTGGTGCAGGTGGCGTTGGAGCTCTCTGATCTCGAAAAGTTCACGGGGCGCAAGGAGCCGACGGTGATCACGTGA
RNAi target sequence in UGPase	TCCAATTCGGACAACCTGGGCGCGACCTTGGATCTGTCCCTGCTCACGTATTTCGCCGAGTCGGGCTCCTCCTTCATGATGGAGTGCGC GGAGAGGACGGAGGCGGACAAGAAGGGGGGGCACCTGGCGGTACGGTCCAGCGACGGCCAGCTGATCTTGCGGGAATCTGCCCAGT GTGCCAAGGAAGACGAACCCGCCTTCCAAGATGTCTCGCGGCACAAGTACTTCAA

**TABLE 2:** Used primers in this study.

Name	Direction	Sequence (5′→ 3′)	Purpose
P1	Fwd	CAAGAAGTCTGTTTTGGAAGCATGGCCAAGTTGACCAGTGC	Amplification of Shble-sense for Gibson assembly
P2	Rev	ACCCCCAACCAATTCTCGCCTGACTTTGAAGTACTTGTGCCGCGA	
P3	Fwd	TGTCTGCGGCACAAGTACTTCAAAGTCAGGGCAGAATGGTTGG	Amplification of Linker+Antisense+TUBt for Gibson assembly
P4	Rev	GGGCCTCTAGATGCTGAGGATCCTCGCTTGTAGCCTCATTTC	
P5	Fwd	GAATGAGGCTATCAGCGAGGATCCTCGAGCATGCATCTAGAGGG	Amplification of pNs_Ri_UGPase vector backbone for Gibson assembly
P6	Rev	AACGGCACTGCAACTTGGCATGCTTCACAAAAAGACAGCTTGATATTGAC	
Q1	Fwd	GTCCCTGCTCACTATTTTGC	qRT-PCR for RNAi target (UGPase)
Q2	Rev	GCAGATTCCGAAGATCAG	
Q3	Fwd	GTGTTTCCCTCCATCGTG	qRT-PCR for Actin
Q4	Rev	CCAGTTCGTCACAATACCG	
McrBC-FWD	Fwd	TGCTATCGAATACCAAAACATTGAAGGC	McrBC-PCR for RNAi target
McrBC-REV	Rev	AACTCCACCACCTTCTTCCT	

For genetic transformation, particle bombardment was performed as previously described (Kang et al., 2015). The pNS_Ri_UGPase was linearized using the BglII restriction enzyme and coated onto microcarrier gold particles (Bio-Rad, Hercules, CA). A mixture containing 25 μL of 50% glycerol-suspended gold particles, 6 μL of concentrated DNA, 50 μL of 2.5 M CaCl2, and 20 μL of 0.1 M spermidine was prepared through vortexing. The DNA-coated gold particles were subsequently washed with 70% ethanol and resuspended in 100% ethanol before use. *N. salina* cells were cultivated to the mid-exponential phase and adjusted to a concentration of 2 × 10? cells. The cells were then layered onto cellulose acetate membrane filters placed on F2N agar plates supplemented with 1 μL/mL ampicillin. Particle bombardment was conducted at a helium pressure of 700 psi with a target distance of 3 cm using a GDS-80 low-pressure gene-delivery system (Wealtec, Sparks, NV, United States). After transformation, cells were released into a modified F2N liquid medium and incubated for 24 h at 23°C under low light conditions (10 μmol photons/m^2^/s). The transformed cells were then harvested via centrifugation at 3,500 rpm for 15 min and plated onto F2N agar plates containing 2.5 μg/mL Zeocin for selection.

### 2.3 McrBC-PCR

Initial screening for RNAi transformants was carried out using McrBC-PCR to assess cytosine methylation within the targeted gene (Wei et al., 2017). The McrBC enzyme selectively cleaves methylated DNA, allowing for the identification of epigenetic modifications induced by RNAi. Genomic DNA was extracted from UGPase-knockdown *N. salina* strains using Instagene Matrix (Bio-Rad, United States) following the manufacturer’s instructions and incubated overnight at 37°C with McrBC (NEB, Boston, MA, United States). PCR was then conducted to confirm the presence of methylated sequences. As a negative control, a parallel reaction was performed using McrBC-digested DNA without GTP, as McrBC enzymatic activity is dependent on GTP as a cofactor.

### 2.4 Quantitative real-time PCR

Quantitative real-time PCR (qRT-PCR) was performed to evaluate UGPase gene expression in wild-type and RNAi-transformed candidate strains. Total RNA was extracted using the NucleoZol reagent (Macherey–Nagel, Germany) following the manufacturer’s instructions. To eliminate residual genomic DNA, the RNA samples were treated with DNA-free DNase (Ambion, United States). Reverse transcription into cDNA was conducted using Superscript III Reverse Transcriptase (Invitrogen, United States) with an oligo(dT)20 primer (Invitrogen, United States).

The qRT-PCR was performed using a CFX96 Real-Time System (Bio-Rad, United States) with primers designed for UGPase (Q1, Q2) and actin (Q3, Q4) as an internal reference (Tables 1, 2). Each 20 μL reaction contained 2 μL of cDNA (corresponding to 20 ng of total RNA), 0.5 μL of each primer (10 μM), 7 μL of distilled water, and 10 μL of Universal SYBR Supermix (Bio-Rad, United States). The PCR conditions included an initial denaturation step at 95°C for 2 min, followed by 40 cycles of 95°C for 10 s, 60°C for 10 s, and 72°C for 20 s. A final denaturation step at 95°C for 10 s was followed by a melting curve analysis from 65°C to 95°C. Gene expression levels were quantified using the 2^–ΔΔ^*^Ct^* method, and statistical significance was determined via Student’s *t*-test (Kang et al., 2017).

### 2.5 Growth analysis

Cell growth was assessed by measuring cell density and dry cell weight (DCW). Cell density was determined using a Cellometer Auto X4 Cell Counter (Nexcelom Bioscience, MA, United States). DCW was measured by filtering 5 mL of culture onto GF/C filter papers (Whatman, Maidstone, United Kingdom), which were then washed twice with deionized water and dried overnight at 105°C before weighing.

### 2.6 Total carbohydrate analysis

Total carbohydrate content was quantified using the anthrone-sulfuric acid method (Leyva et al., 2008). A 5 mg sample of lyophilized biomass was resuspended in 1 mL of deionized water and mixed thoroughly. The solution was then reacted with anthrone reagent (2 mg anthrone in 75% v/v sulfuric acid) at 100°C for 15 min. After cooling on ice for 5 min, absorbance was measured at 620 nm using a glucose standard curve (0–240 mg/L) (Shin et al., 2024).

### 2.7 Total lipid analysis

Lipid extraction was performed following the Folch method (Folch et al., 1957). Briefly, 20 mg of lyophilized biomass was mixed with 10 mL of a chloroform-methanol solution (2:1 v/v) and sonicated at 25°C for 1 h. Following the addition of 2.5 mL of deionized water, the mixture was vortexed vigorously for 5 min and centrifuged to separate the organic phase, which was filtered through a 0.20 μm RC-membrane syringe filter (Sartorius Stedim Biotech, Germany). Lipid content was quantified by drying the extracted lipids on pre-weighed aluminum dishes and calculated using the following formula:


$$Totallipidcontent\left(\%\right)=\frac{\left(\mathrm{WL}-\mathrm{WD}\right)\,\mathrm{VC}}{\mathrm{VP}\,\mathrm{WS}}\times 100$$

where W_*L*_ and W_*D*_ denote the weight of the aluminum dish with and without lipid, W_*S*_ is the biomass weight, V_*C*_ is the total chloroform volume, and V_*P*_ is the transferred chloroform volume.

### 2.8 Fatty acid methyl ester analysis

Fatty acid methyl ester (FAME) analysis was conducted following a previously established protocol (Jeon et al., 2021). Fifty milliliters of culture were harvested by centrifugation at 4,000 rpm for 15 min, washed with deionized water, and lyophilized for 3 days. To extract total lipids from the lyophilized biomass, a modified Folch method (Folch et al., 1957) was employed. The dried biomass was resuspended in a chloroform-methanol solution (2:1, v/v) to facilitate lipid extraction. The mixture was vigorously vortexed for 10 min to ensure complete dissolution. To quantify extracted lipids, heptadecanoic acid (C17:0) was added as an internal standard at a concentration of 0.5 mg. For transesterification, 1 mL of methanol and 300 μL of sulfuric acid were added to the lipid extract, and the reaction mixture was heated at 100°C for 20 min. The final FAME samples were analyzed using a gas chromatograph (HP 6890, Agilent Technologies, CA, United States) equipped with a flame ionization detector (FID). Separation was achieved using an HP-INNOWax polyethylene glycol column (HP 19091N-213, Agilent Technologies, CA, United States).

### 2.9 Statistical analysis

Statistical significance was determined using Student’s *t*-test. An F-test was first performed to assess variance equality between groups. If the variances were equal (*p* > 0.05), a standard Student’s *t*-test was applied. If the variances were unequal (*p* ≤ 0.05), Welch’s correction was used. Differences were considered statistically significant at *p* < 0.05 (*), highly significant at *p* < 0.01 (**), and extremely significant at *p* < 0.001 (***).

## 3 Results and discussion

### 3.1 The design of RNAi vector for UGPase knockdown and mutant generation

RNA interference (RNAi) is a gene silencing mechanism that operates at both post-transcriptional (PTGS) and transcriptional (TGS) levels. While PTGS involves direct degradation of target mRNA via small interfering RNAs (siRNAs) and the RNA-induced silencing complex (RISC), TGS is associated with epigenetic modifications, such as cytosine DNA methylation and histone modifications, which repress gene transcription. In plants, TGS is often triggered by small RNAs and involves RNA-directed DNA methylation (RdDM), a process in which siRNAs recruit Argonaute (AGO) proteins to complementary genomic loci, leading to the recruitment of DNA methyltransferases that modify cytosine residues (Chan, 2008, Zilberman et al., 2003, Wei et al., 2017). This pathway effectively suppresses gene expression by blocking transcription initiation or elongation.

To determine whether *Nannochloropsis salina* possesses the necessary components for RNAi-mediated gene silencing via methylation, we performed domain analysis of key RNAi machinery proteins: Argonaute (AGO), Dicer, and RNA-dependent RNA polymerase (RDR). As shown in Supplementary Figure 2, our analysis confirmed the presence of these core RNAi components in *N. salina*, suggesting that this microalga is capable of RNAi-induced transcriptional repression via DNA methylation.

To suppress UGPase expression, the pNs_Ri_UGPase, designed to generate a hairpin RNA (hpRNA) upon transcription, was introduced into N. salina (Figure 1A). The expressed hpRNA was processed into double-stranded RNA (dsRNA), which was subsequently cleaved by Dicer into small interfering RNAs (siRNAs), leading to targeted silencing of the UGPase gene. To determine whether RNAi-induced methylation occurred at the UGPase locus, we employed McrBC-PCR screening (Wei et al., 2017; Figures 1B, C). The McrBC enzyme specifically cleaves methylated DNA in the presence of GTP, leading to reduced PCR amplification when methylation is present. To identify transformants with active RNAi, genomic DNA was treated with McrBC, followed by PCR amplification using McrBC-FWD and McrBC-REV primers (Table 2). As shown in Figure 1C, transformants treated with GTP exhibited fainter or absent PCR bands, indicating the presence of cytosine methylation at the UGPase locus. These methylation-positive transformants were selected as first-round RNAi candidates for further validation. The UGPase knockdown transformants were further validated by qRT-PCR targeting the UGPase transcript (Figure 1D). The qRT-PCR results revealed a significant reduction in UGPase transcript abundance in RNAi transformants compared to wild-type N. salina. This strongly supports that RNAi-mediated knockdown of UGPase was successfully achieved, likely through both PTGS (mRNA degradation) and TGS (DNA methylation-induced transcriptional repression).

In *Nannochloropsis* and other heterokont microalgae, chrysolaminarin serves as the primary carbohydrate storage molecule. This β-1,3-glucan polysaccharide is synthesized and accumulated in the cytoplasm, functioning as a crucial energy reserve (Wang et al., 2014, Daboussi et al., 2014). The biosynthesis of chrysolaminarin is directly linked to UGPase, a key enzyme that catalyzes the production of UDP-glucose, a fundamental precursor in the chrysolaminarin synthesis pathway. Downregulation of UGPase is expected to disrupt this metabolic flux, leading to a reduction in total carbohydrate content while potentially enhancing alternative carbon allocation, such as lipid biosynthesis (Hildebrand et al., 2017).

To screen the transformants for further studies, total carbohydrate content was measured in the McrBC-PCR and qRT-PCR validated transformants (Supplementary Figure 3). Among the transformants, strains 5 and 26 exhibited a reduction in total carbohydrate content. Based on these results, these two strains were selected to investigate the metabolic effects of UGPase knockdown and assess potential growth enhancement and lipid accumulation as a compensatory response to reduced carbohydrate synthesis. These two transformants were designated as NsRiUGPase 5 and NsRiUGPase 26 for subsequent analyses.

### 3.2 Growth performance and carbon partitioning in UGPase RNAi mutants

**FIGURE 2:**
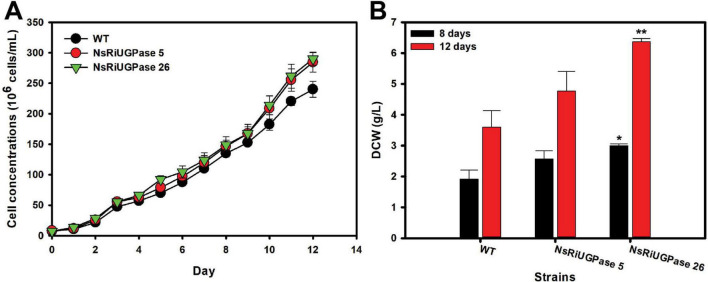
Growth performance of *N. salina* wild-type (WT) and the NsRiUGPase transformants. **(A)** Growth curves of WT and RNA interference (RNAi) transformants (NsRiUGPase 5 and NsRiUGPase 26) were measured as cell concentration (cells/mL) over time under photoautotrophic conditions. **(B)** Dry cell weight (DCW) of WT, NsRiUGPase 5, and NsRiUGPase 26 at days 8 and 12 of cultivation. Dry cell weight (DCW) was determined by filtering and drying biomass. All data represent the mean ± standard error (*n* = 3). Significant differences against WT for the same conditions and same time points, as determined by Student’s *t*-test, are indicated by asterisks (*p < 0.05, **p < 0.01, ***p < 0.001).

The RNAi-mediated knockdown of UGPase in *Nannochloropsis salina* led to a significant improvement in growth performance compared to the wild-type (WT), as evidenced by both cell concentration and dry cell weight (DCW) measurements (Figure 2). On day 8, the WT strain reached a cell concentration of 135.0 × 10^6^ cells/mL, while the NsRiUGPase 5 and 26 exhibited slightly higher values of 146.3 × 10^6^ and 148.7 × 10^6^ cells/mL, corresponding to increases of 8.3% and 10.1%, respectively. On day 12, this difference became more apparent. The WT reached 240.0 × 10^6^ cells/mL, whereas mutant 5 and mutant 26 reached 284.3 × 10^6^ and 290.0 × 10^6^ cells/mL, representing 18.5% and 20.8% higher concentrations than WT (Figure 2A). These observations suggest that UGPase knockdown may promote sustained cell proliferation, potentially extending the exponential growth phase.

A similar but more pronounced trend was observed in DCW measurements (Figure 2B). On day 8, DCW increased by 33.9% in the NsRiUGPase 5 (2.57 g/L) and 56.2% in the NsRiUGPase 26 (3.00 g/L) compared to WT (1.92 g/L). On day 12, the WT reached 3.60 g/L, whereas mutant 5 reached 4.77 g/L (32.4% higher), and mutant 26 reached 6.37 g/L (76.9% higher). Notably, the greater increase in DCW compared to cell concentration suggests that the RNAi mutants not only proliferated more but also accumulated more intracellular biomass.

**FIGURE 3:**
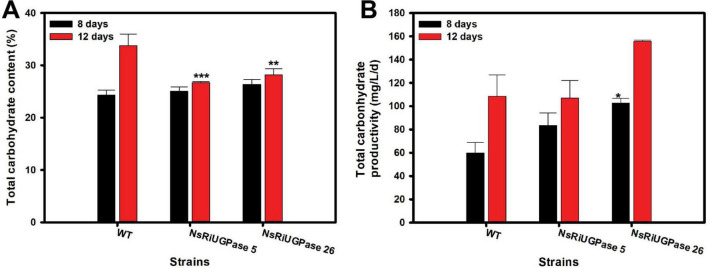
Carbohydrate analysis of *N. salina* wild-type (WT) and the NsRiUGPase transformants. **(A)** Total carbohydrate content of WT, NsRiUGPase 5, and NsRiUGPase 26 at days 8 and 12 of cultivation. **(B)** Total carbohydrate productivity (g/L/day) of WT, NsRiUGPase 5, and NsRiUGPase 26 at days 8 and 12. All data represent the mean ± standard error (*n* = 3). Significant differences against WT for the same conditions and same time points, as determined by Student’s *t*-test, are indicated by asterisks (*p < 0.05, **p < 0.01, ***p < 0.001).

To better understand how these growth changes were linked to metabolic shifts, we examined intracellular carbohydrate accumulation (Figure 3). Carbohydrate content, expressed as a percentage of biomass, showed patterns consistent with initial screening results (Figure 3A). On day 8, carbohydrate levels were similar across all strains, with WT at 24.28% biomass, mutant 5 at 25.02% biomass, and mutant 26 at 26.32% biomass. On day 12, however, the mutant showed lower carbohydrate contents than WT. Compared to WT (33.8% biomass), carbohydrate content decreased by 20.9% in mutant 5 (26.7% biomass), and 16.7% in mutant 26 (28.2% biomass), indicating a shift in carbon allocation away from carbohydrate storage in the later growth phase.

Despite this decrease in carbohydrate content, carbohydrate productivity showed a different pattern (Figure 3B). On day 8, the productivity was markedly higher in the mutants, with mutant 5 reaching 88.44 mg/L/day (39.3% higher than WT) and mutant 26 reaching 102.58 mg/L/day (71.2% higher) compared to WT (63.52 mg/L/day). Even on day 12, when carbohydrate content had declined, carbohydrate productivity remained stable or increased. Mutant 5 showed a slight decrease of 1.4% (107.05 mg/L/day), while mutant 26 exhibited a 43.4% increase (155.65 mg/L/day) compared to WT (108.52 mg/L/day). These results suggest that although the mutants accumulated less carbohydrate relative to their biomass, the total carbohydrate production was maintained or enhanced due to the substantial increase in biomass.

A possible explanation for these findings is that UGPase knockdown altered carbohydrate storage dynamics, potentially influencing carbon allocation away from chrysolaminarin synthesis (Hildebrand et al., 2017). In WT cells, as growth slows, carbon from photosynthesis is typically redirected toward carbohydrate storage, coinciding with the transition from exponential to stationary phase. However, in the RNAi mutants, reduced UGPase activity may have limited the capacity to synthesize and store chrysolaminarin, allowing more carbon to remain available for immediate cellular processes. This shift in carbon metabolism could have contributed to sustained active proliferation and increased biomass accumulation. Although there are no reports of enhanced growth resulting from UGPase knockdown, several studies have shown that excessive carbon storage due to UGPase overexpression can negatively affect growth. For example, overexpression of Ugp from *Acetobacter xylinum* in *Populus alba × grandidentata* led to increased soluble sugar levels but was accompanied by reduced plant height, stem diameter, and leaf size (Coleman et al., 2007). Similarly, *OsUgp1* overexpression in *Oryza sativa* (rice) increased sucrose content but resulted in pronounced biomass reduction and a dwarf phenotype (Zhang et al., 2023). These observations suggest that an imbalance in carbon storage can limit growth across species (Zhang, 2025). Thus, in *N. salina*, partial suppression of UGPase might have contributed to improved growth and biomass accumulation by maintaining a more favorable balance in carbon utilization. Based on these observations, we hypothesized that the altered carbon allocation in the RNAi mutants might also influence lipid biosynthesis (Yang et al., 2022, Yang et al., 2019, Huang et al., 2018). Therefore, we further investigated lipid content and productivity to determine whether this metabolic shift was reflected in lipid accumulation.

### 3.3 Lipid accumulation and composition in RNAi mutants

**FIGURE 4:**
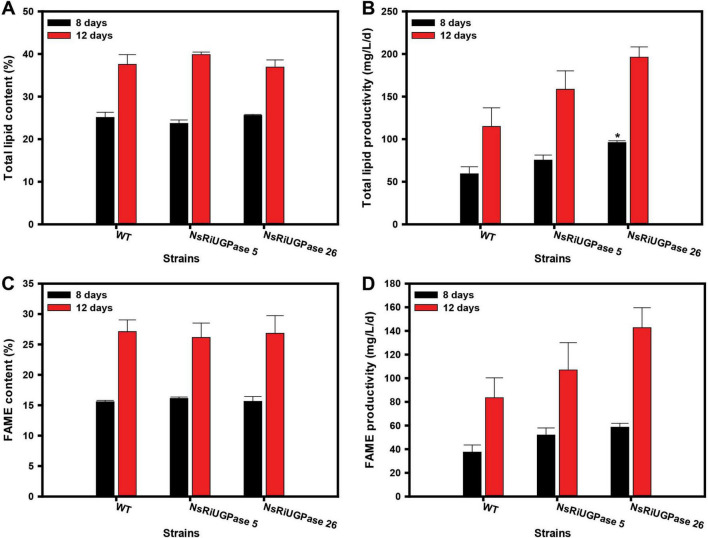
Lipid and fatty acid methyl ester (FAME) analysis in *N. salina* wild-type (WT) and the NsRiUGPase transformants. **(A)** Lipid content and **(B)** lipid productivity of WT, NsRiUGPase 5, and NsRiUGPase 26 at days 8 and 12 of cultivation, determined by the gravimetric method (Folch extraction). **(C)** FAME content and **(D)** FAME productivity were measured on days 8 and 12 and analyzed by gas chromatography (GC) following transesterification. All data represent the mean ± standard error (*n* = 3). Significant differences against WT for the same conditions and same time points, as determined by Student’s *t*-test, are indicated by asterisks (*p < 0.05, **p < 0.01, ***p < 0.001).

Lipid accumulation was assessed to determine whether the metabolic shift observed in RNAi mutants influenced lipid biosynthesis and productivity (Figure 4). Despite the significant differences in growth and biomass accumulation (Figure 2), total lipid content remained largely unchanged among WT and RNAi mutants, with variations within 6% at both time points. Neither mutant exhibited a statistically significant difference from WT in lipid content on day 8 or day 12. However, due to the increased biomass in the RNAi mutants, lipid productivity was substantially higher. On day 8, the NsRiUGPase 5 showed a 27.1% increase (75.3 mg/L/day) compared to WT (59.3 mg/L/day), while the NsRiUGPase 26 exhibited a 61.9% increase (96.0 mg/L/day). This trend persisted on day 12, where WT reached 114.8 mg/L/day, whereas mutant 5 showed a 38.0% increase (158.5 mg/L/day) and mutant 26 displayed a 71.0% increase (196.3 mg/L/day). Furthermore, both RNAi mutants exhibited higher lipid and FAME contents per cell than the WT (Supplementary Figure 4), aligning with their enhanced productivity pattern. These results suggest that the improvement in lipid productivity was attributed to the combined effects of increased biomass and lipid accumulation.

Total lipid analysis provides a comprehensive measure of all cellular lipids, including triacylglycerols (TAGs), phospholipids, glycolipids, and free fatty acids (A. Patel et al., 2019). While this method is useful for assessing lipid accumulation at the cellular level, it does not distinguish between lipid classes and thus includes lipid fractions that are not suitable for biodiesel production. To gain a more detailed understanding of lipid composition, transesterification followed by gas chromatography (GC) was performed to analyze fatty acid methyl esters (FAMEs), which are more directly relevant to biodiesel applications.

The FAME content followed a similar trend to total lipid content, with no significant differences among WT and RNAi mutants on either day 8 or day 12. The FAME content in all three strains remained within 4% of each other, indicating that UGPase knockdown did not significantly alter the proportion of lipids that could be converted into biodiesel precursors. However, the absolute FAME content was lower than the total lipid content. On day 8, while total lipid content was 24.8 ± 0.8%, FAME content was 15.8 ± 0.2%, accounting for approximately 63% of total lipids. Similarly, on day 12, total lipid content reached 38.1 ± 1.3%, whereas FAME content was 26.7 ± 0.4%, representing about 70% of total lipids. This suggests that a substantial fraction of cellular lipids, including phospholipids, glycolipids, and sterols, were not converted into biodiesel-relevant FAMEs. The fact that these proportions were consistent across WT and mutants implies that UGPase knockdown did not influence the relative distribution of lipid classes.

To examine whether UGPase suppression influenced fatty acid composition, FAME profiles were analyzed using GC (Supplementary Figure 5). The overall fatty acid profiles were similar between WT and RNAi mutants, indicating that UGPase down-regulation did not significantly affect fatty acid composition. However, temporal changes were observed across all strains: on day 8, palmitoleic acid (C16:1) and eicosapentaenoic acid (EPA, C20:5) were predominant (27%–30%), whereas by day 12, EPA levels decreased (12%–13%) and palmitic acid (C16:0) increased (31%). This shift is consistent with lipid remodeling during cultivation (Koh et al., 2024) and was not specifically associated with UGPase knockdown. These results suggest that while UGPase knockdown enhanced lipid productivity, it did not alter the overall balance among saturated fatty acids, monounsaturated fatty acids, and polyunsaturated fatty acids in the mutants.

## 4 Conclusion

This study demonstrated that RNAi-mediated suppression of UGPase in *Nannochloropsis salina* led to measurable changes in biomass accumulation and lipid productivity without altering lipid composition. The knockdown of UGPase resulted in reduced carbohydrate accumulation, which coincided with prolonged cell proliferation and increased overall biomass. A reduction in chrysolaminarin synthesis is generally thought to increase carbon flux toward lipid accumulation. However, our findings suggest that rather than increasing lipid content per cell, the diverted carbon was primarily utilized for biomass production and active metabolism, potentially sustaining growth for a longer period. While total lipid content remained largely unchanged, the substantial increase in biomass led to higher lipid productivity in the RNAi mutants compared to WT. These findings demonstrate that the downregulation of UGPase via RNAi can influence biomass and lipid productivity in *Nannochloropsis*, suggesting a potential strategy for metabolic engineering in microalgae.

## Data Availability

The datasets presented in this study can be found in online repositories. The names of the repository/repositories and accession number(s) can be found in the article/Supplementary material.
