# Supplementary material for: Enhancing lipid production in Nannochloropsis salina via RNAi-mediated downregulation of carbohydrate biosynthesis
Source: Front Microbiol. 2025 May 21;16:1601691. doi: 10.3389/fmicb.2025.1601691 (PMC12133928; doi:10.3389/fmicb.2025.1601691)
Supplement: Supplementary file 1 [file Data_Sheet_1.docx]

Supplementary Material


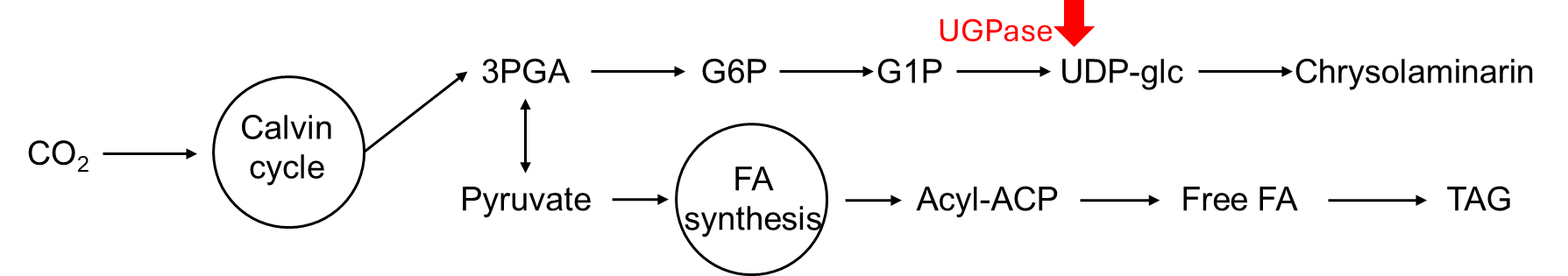


**Supplementary Figure S1.** Schematic representation of TAG and chrysolaminarin biosynthetic pathways. In this study, UGPase expression was knocked down via RNAi to suppress chrysolaminarin synthesis and redirect carbon flux toward enhanced lipid (TAG) accumulation. 3PGA, 3-phosphoglycerate; ACP, acyl carrier protein; FA, fatty acids; G1P, glucose-1-phosphate; G6P, glucose-6-phosphate; TAG, triacylglycerol; UDP-Glc, uridine diphosphate glucose; UGPase, UDP-glucose pyrophosphorylase.


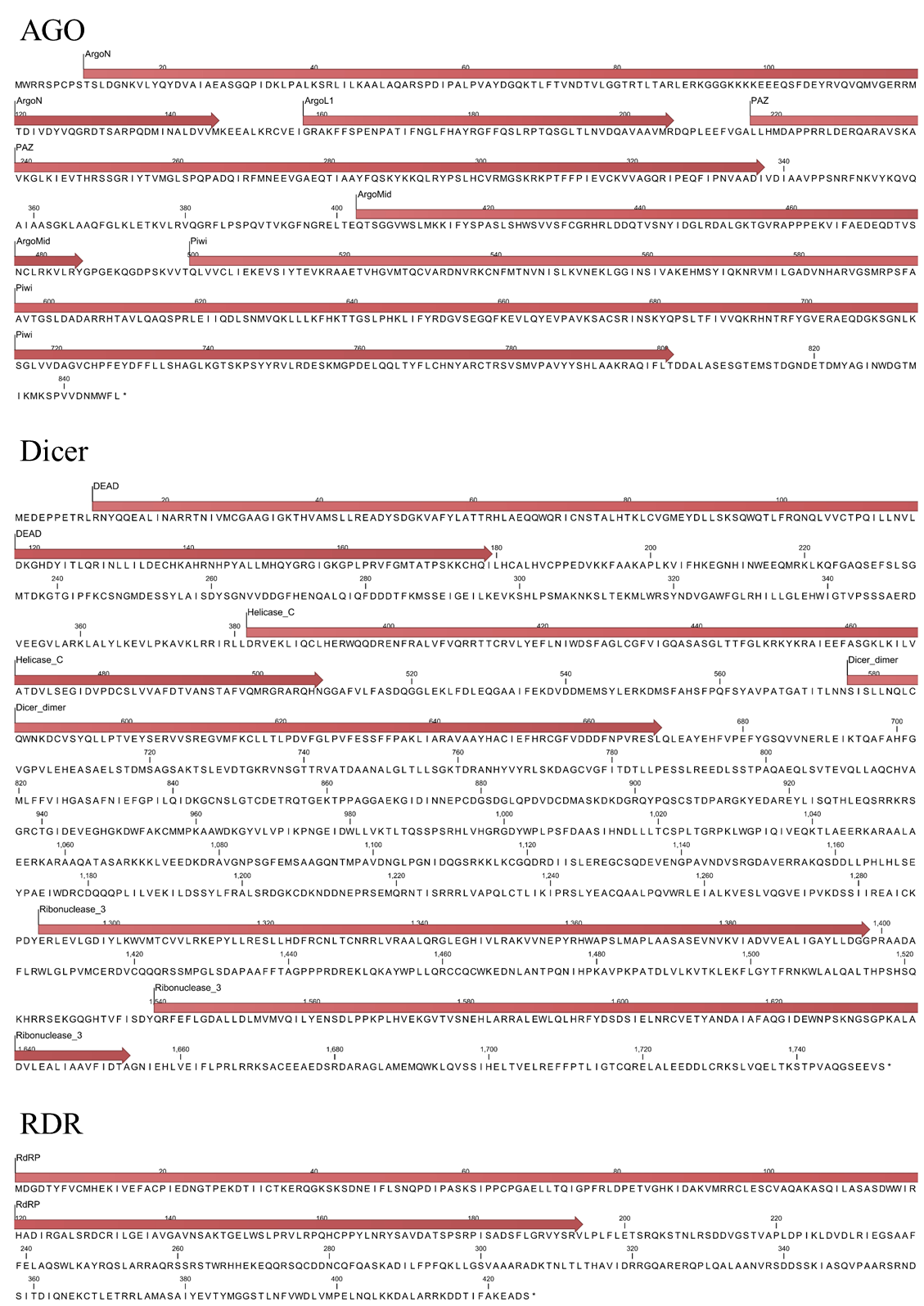


Supplementary Figure S2. Features of the three key components in RNAi machinery in Nannochloropsis salina CCMP5776

**Supplementary Figure S3.** Total carbohydrate content analysis of candidate strains. Cells were cultured in F2N medium under a light intensity of 120 μmol photons/m²/s at 25°C with 3% CO₂ and shaking at 250 rpm. Total carbohydrate content was measured on day 12.


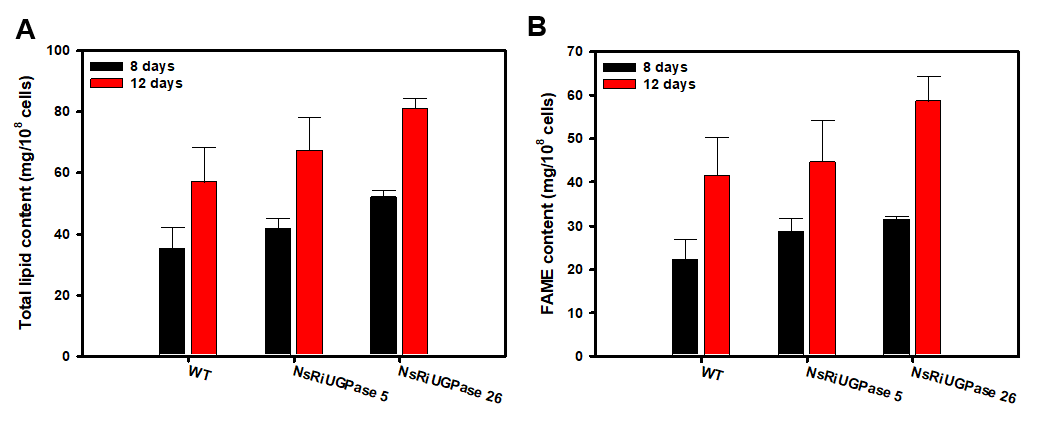


**Supplementary Figure S4.** Lipid and fatty acid methyl ester (FAME) analysis in *N. salina* WT and the NsRiUGPase transformants normalized by cell concentration. **(A)** Lipid content and **(B)** FAME content per 10^8^ cells were analyzed on day 8 and 12. All data represent the mean ± standard error (*n* = 3). Significant differences against WT for the same conditions and same time points, as determined by Studentʼs *t* test, are indicated by asterisks (*p < 0.05, **p < 0.01, ***p < 0.001).


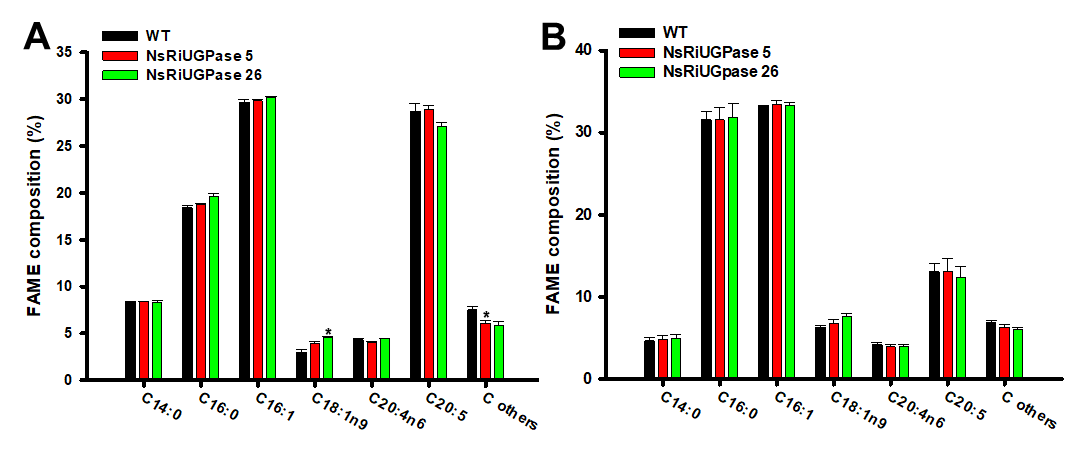


**Supplementary Figure S5.** Fatty acid methyl ester (FAME) composition in wild-type and RNAi transformants. FAME profiles of WT, NsRiUGPase 5, and NsRiUGPase 26 strains on day 8 (A) and day 12 (B). Cells were cultured in F2N medium under a light intensity of 120 μmol photons/m²/s at 25°C with 3% CO₂ and shaking at 250 rpm. All data represent the mean ± standard error (*n* = 3). Significant differences against WT for the same conditions and same time points, as determined by Studentʼs *t* test, are indicated by asterisks (*p < 0.05, **p < 0.01, ***p < 0.001).
